# Distribution Characteristics of High-Background Elements and Assessment of Ecological Element Activity in Typical Profiles of Ultramafic Rock Area

**DOI:** 10.3390/toxics13070558

**Published:** 2025-06-30

**Authors:** Jingtao Shi, Junjian Liu, Suduan Hu, Jiangyulong Wang

**Affiliations:** Langfang Natural Resources Comprehensive Survey Center, China Geological Survey, Langfang 065000, China; sjt969818@163.com (J.S.); langyuanxianpa@live.cn (J.W.)

**Keywords:** ultramafic rock, high-background elements, element migration, ecological element assessment, tessier speciation analysis, mining disturbance

## Abstract

This study investigates the weathering crust composite of serpentine, pyroxenite and granite in the Niangniangmiao area, the weathering crusts inside and outside the mining area were compared respectively, systematically revealing the distribution patterns, migration pathways, and ecological element activity characteristics of high-background elements (e.g., chromium (Cr) and nickel (Ni)) through precise sampling, the Tessier five-step sequential extraction method, and a migration coefficient model. Key findings include: (1) Element distribution and controlling mechanisms: The average Cr and Ni contents in the serpentinite profile are significantly higher than those in pyroxenite. However, the semi-weathered pyroxenite layer exhibits an inverted Cr enrichment ratio in relation to serpentinite, 1.8× and 1.2×, respectively, indicating that mineral metasomatic sequences driven by hydrothermal alteration dominate element differentiation; the phenomenon of inverted enrichment of high-background elements occurs in the weathering crust profiles of the two basic rocks. (2) Dual impacts of mining activities on heavy metal enrichment: Direct mining increases topsoil Cr content in serpentinite by 40% by disrupting parent material homology, while indirect activities introduce exogenous Zn and Cd (Spearman correlation coefficients with Cr/Ni are from ρ = 0.58 to ρ = 0.72). Consequently, the bioavailable fraction ratio value of Ni outside the mining area (21.14%) is significantly higher than that within the area (14.30%). (3) Element speciation and ecological element activity: Over 98% of Cr in serpentine exists in residual fractions, whereas the Fe-Mn oxide-bound fraction (F3) of Cr in extra-mining pyroxenite increases to 5.15%. The element activity in ecological systems ranking of Ni in soil active fractions (F1 + F2 = 15%) follows the order: granite > pyroxenite > serpentine. Based on these insights, a scientific foundation for targeted remediation in high-background areas (e.g., prioritizing the treatment of semi-weathered pyroxenite layers) can be provided.

## 1. Introduction

Mining has always been an indispensable driving force for socioeconomic development. However, resource extraction is often accompanied by ecological and environmental problems, which have shown a trend of increasing severity. The persistent impacts of heavy metals, particularly on soil, water bodies, and ecosystems, have garnered widespread attention [1,2,3,4]. Among various mineral resources, ultrabasic rocks (such as serpentine and pyroxenite) rich in strategic metals like Ni, Cr, and Co provide important basic materials for industrial production. With the rapid advancement in new energy technologies and surging demand from related industries, the global appetite for critical metals such as Ni, Cr, and Co has intensified across China, the Americas, Europe, and beyond. Consequently, serpentine—once overlooked for decades in Europe—is now witnessing a renewed exploration boom [5,6]. However, we must recognize that as members of the “heavy metal” category, these metals inherently possess distinct metallic characteristics typical of their group [7,8]. It is highly necessary to assess their distribution patterns, migration pathways, and ecological element activity during the development of ore deposits.

Soils derived from the weathering of ultramafic rocks (e.g., serpentine soils) exhibit distinctive geochemical properties, involving an elevation in the Mg/Ca ratio, nutrient scarcity, and the natural concentration of nickel, chromium, and cobalt [9,10,11]. As both a vast repository of metallic resources and a ‘natural laboratory’ for investigating heavy metal migration and biogeochemical processes, ultramafic-derived soils offer unique insights. The mobility and bioavailability of heavy metals during ultramafic rock pedogenesis are governed by multiple factors, including host mineralogy (e.g., olivine, serpentine, chromite), pedogenic age, soil pH, organic carbon content, and clay mineral assemblage [12,13,14]. For instance, weathering-resistant chromite and iron oxides, which host higher Cr concentrations, exhibit lower mobility; whereas Ni is preferentially released from primary silicates (e.g., olivine) and sorbs onto or complexes with secondary clay minerals such as montmorillonite and Fe-Mn (oxy)hydroxides [15,16]. Furthermore, across climatic zones (e.g., temperate vs. tropical), variations in pedogenesis rates and secondary mineral formation act as key drivers of heavy metal retention or release [17,18].

Human activities have exacerbated heavy metal migration, with extensive mining practices posing a significant threat to environmental safety. Historical open-pit mines, tailings impoundments, and exploration wells have become primary ‘sources’ of heavy metal dispersion, enabling their transport via fluvial pathways (e.g., river sediments) and aeolian processes (e.g., dust) into neighboring agricultural lands and ecosystems [19,20,21]. For instance, Ni concentrations in soils adjacent to abandoned Ni mines in southern Czechia have reached up to 4950 mg/kg, surpassing the local regulatory thresholds for agricultural land [22]. Soil Cu contamination in Baotou Iron Mining Area, China, is primarily derived from tailings and shows a significant spatial correlation with mining transportation corridors [23]. Moreover, fluctuations in redox conditions—such as the flooding–drainage cycles in paddy soils—can induce the conversion of Cr(III) to toxic Cr(VI), enabling its migration via dissolution–reprecipitation of Fe/Mn (oxy)hydroxides [24].

Previous studies have predominantly focused on the control mechanisms of heavy metal material types during the pedogenesis of ultrabasic rocks (e.g., primary mineralogical differences between serpentinite and pyroxenite), but generally overlooked the reconstructive effect of hydrothermal alteration on metal occurrence states and the spatial differentiation patterns of heavy metal active species under anthropogenic disturbances (e.g., mining activities). This study for the first time reveals that: ① hydrothermal alteration can cause the Cr content in the semi-weathered layer of gabbro to increase by 1.8 times relative to the background value, forming a “heavy metal inverted enrichment” phenomenon independent of primary lithology; ② the active Ni content in the granite area reaches 19.3% of that in typical ultrabasic rock areas, revised the traditional understanding that “material composition is the dominant determinant of environmental impact mechanisms”. These findings have transcended the traditional management paradigm centered on “primary lithology classification” for geological resource regulation and established a dual-factor-driven heavy metal migration model of “hydrothermal alteration-anthropogenic disturbance”.

Taking the Niangniangmiao serpentinite mining area as the research object, this study aims to: ① verify the universality of the “hydrothermal alteration-guided heavy metal inverted enrichment” model; ② quantify the impact of mining activities on the spatial distribution of Cr/Ni active species; ③ propose differentiated restoration strategies based on risk classification. The core hypothesis is that hydrothermal alteration leads to non-primary mineral enrichment of Cr in the semi-weathered layer of gabbro, while the distribution of active Ni is controlled by the coupling of “lithological material basis-mining disturbance intensity”, in which anthropogenic activities in the granite area can significantly enhance the bioavailability of Ni. This research framework not only fills the theoretical gap in ecological element activity assessment of hydrothermally modified deposits but also provides a new methodology for heavy metal element control in “natural-anthropogenic” dual-disturbance areas.

## 2. Study Area Setting

**Figure 1:**
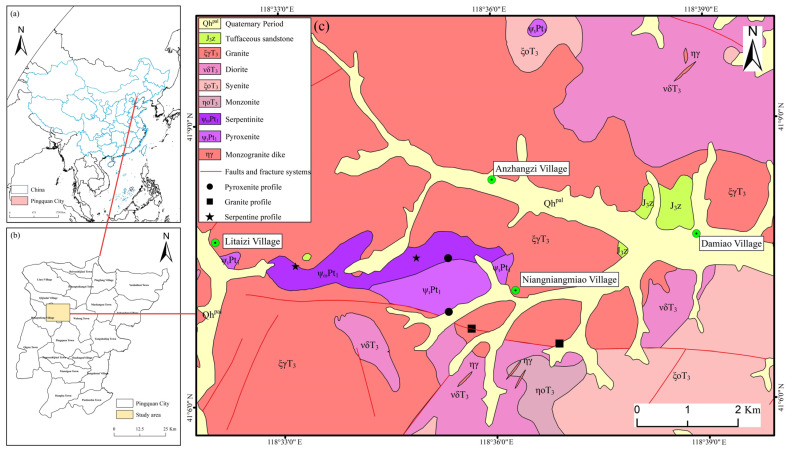
Geographical and geological setting of the study area: (**a**) Worldwide geographical location of China and Pingquan city; (**b**) Geographical location of Pingquan city in Hebei province; (**c**) Simplified geological map of the study area with the locations of the sampling profiles (modified from the 1:50,000 geological map by the Fifth Team, Hebei Regional Geological and Mineral Survey Institute).

Characterized by an annual mean sunshine duration of 2400 h to 3100 h and mean annual precipitation of 400 mm to 800 mm, the study area exhibits relatively low rainfall. The mean temperature falls below 3 °C in January and ranges from 18 °C to 27 °C in July, showcasing distinct seasonal temperature variations. It is categorized as a temperate continental monsoon climate (Köppen: Dwa/Dwb), located in the northeasternmost corner of Hebei Province, China, within the Yanshan Hills—a mountainous region characterized by rolling terrain. Ultramafic rocks, mainly serpentinite (metamorphic rock derived from metamorphosed peridotite) and pyroxenite, outcrop in the Niangniangmiao-Litaizi area, forming an E–W trending belt spanning 2.5 km^2^ (serpentinite) and 1.8 km^2^ (pyroxenite). These are intruded by Late Triassic granitoids and intermittently overlain by Quaternary alluvial gravels and loess deposits (Figure 1). The geological framework of the study area is characterized by Quaternary loose sediments and Mesozoic sedimentary-intrusive complexes. Among them, the Quaternary loose sediments consist of Holocene alluvial–proluvial gravel, sand, and sandy loam, primarily deposited in wider river valleys and floodplains. The sedimentary strata are represented by tuffaceous sandstones of the Late Jurassic Zhangjiakou Formation, while the vein rocks are monzogranite dikes. Based on contact relationships and isotopic dating results, the intrusive rocks in the area are classified into two magmatic epochs: Paleoproterozoic and Triassic. This stratigraphic–magmatic framework provides a basis for understanding the regional tectonic evolution and mineralization potential (Table 1).

## 3. Materials and Methods

### 3.1. Distribution of Sampling Sites and Sample Collection

Mining activities in this area commenced in the late 20th to early 21st century. The ore deposits are predominantly composed of pyroxenite, resulting in the formation of large-scale mining pits. Surrounding these pits are numerous tailings storage facilities, primarily situated in gully systems along piedmont slopes. Over the past decade, agricultural tillage activities and rainfall infiltration processes have driven the migration of tailings discharge fluids toward valleys and surrounding cultivated lands, leading to an upward trend in the occurrence amounts of heavy metals elements in some areas [25,26,27,28], and has modified the compositional framework of the original weathering crust outside the mining pit.

**Figure 2:**
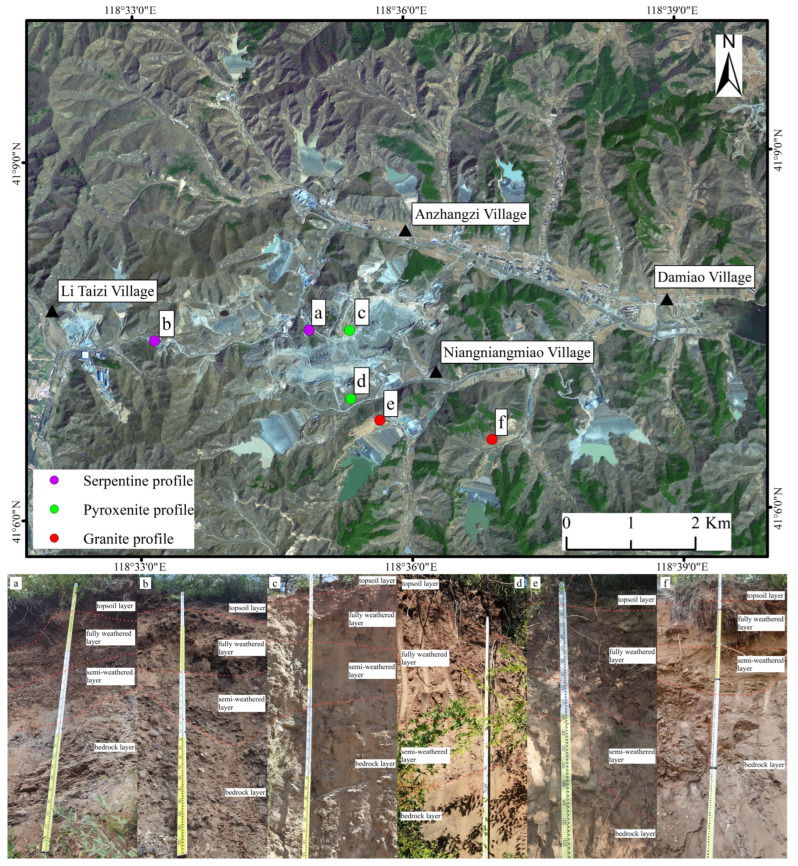
Remote sensing imagery (Landsat-9 imagery) and sampling profile photographs (**a**–**f**) of the study area.

To investigate the distribution of heavy metals in weathering crusts inside and outside the mining area, two weathering profiles (serpentinite and pyroxenite) were established within the mining pit, and one granite weathering profile—unaffected by tailings activities—was sampled at the crest of a tailings reservoir adjacent to the east–west axis of the pit as inner-mining profiles. For outer-mining profiles, three weathering profiles (serpentinite, pyroxenite, and granite) were selected at sites including the roadside of transport routes and the base of tailings reservoirs (Figure 2). The remote sensing imagery dataset was obtained from the Geospatial Data Cloud Platform, using Landsat-9 imagery (https://www.gscloud.cn/ (accessed on 1 May 2025)). The data was acquired in April 2024 with a spatial resolution of 15 m. Sampling was conducted in August, with profile depths varying across sites. Stratified sampling included: one sample from the topsoil layer (0 m to 0.4 m), three samples from the fully weathered layer (0.4 m to 1.4 m), three samples from the semi-weathered layer (1.4 m to 2.3 m), and one fresh bedrock sample. A total of 48 samples were collected from six profiles, all following standardized protocols. Samples were labeled sequentially and transported to the laboratory for analysis.

For analytical procedures, the Analytical Methods for Regional Geochemical Samples (DZ/T 0279-2016) [29] and the Rock and Mineral Analysis [30] were followed to determine As, Cd, Cr, Ni, Cu, Pb, Hg, Zn, CaO, MgO, K_2_O, Na_2_O, Fe_2_O_3_, Al_2_O_3_, SiO_2_, TiO_2_, and Cr/Ni speciation. Analytical methods and detection limits for profile sample parameters are listed in Table 2. The methods listed in the table exhibited precision (RSD) below 5% for both heavy metals and major elements, with standard addition recovery rates ranging from 80% to 120%, which confirms the reliability of the research data.

Heavy metal speciation was analyzed using the Tessier five-step sequential extraction procedure, with operational steps detailed in Table 3, in this study, heavy metal speciation was determined using the Tessier five-step sequential extraction procedure [31,32,33], heavy metals in soils occur in five fractions: exchangeable (F1) refers to the form of heavy metals bound to surface adsorption sites and ion exchange sites in soils or sediments, carbonate-bound (F2) refers to the form of heavy metals combined with carbonate minerals in soils and sediments, Fe/Mn oxide-bound (F3) refers to the form of heavy metals combined with Fe-Mn (hydr)oxides through physical adsorption, chemical coprecipitation, or surface complexation in soils and sediments, organic-bound (F4) refers to the form of heavy metals combined with organic matter through coordination bonds, hydrogen bonds, or hydrophobic interactions in soils and sediments, and residual (F5) refers to the form of heavy metals tightly bound to mineral lattices or encapsulated within minerals in soils and sediments. F5 is determined by decomposing the residue after F1, F2, F3, and F4 with hydrofluoric acid, instead of subtracting the sum of the first four forms from the total amount. The total sum of all forms should be no less than 80% and no more than 105%. If the total sum of all forms exceeds the total amount by more than 5%, the cause should be checked until the rework is completed. All results complied with quality assurance criteria, ensuring reliable data integrity.

During laboratory testing of samples, parallel experiments are conducted for each group. The experimental results are expressed as the average value, and the error of parallel experiments is less than 5%, ensuring that the detection results meet the accuracy requirements.

### 3.2. Evaluation Methods

#### 3.2.1. Migration Coefficient Method

In the calculation of heavy metal migration coefficients, inert elements (e.g., Sc, Zr, Ti, Al) are selected as stable reference elements. In this work, Ti is used as the reference element. The mathematical expression is as follows:

(1) $$i^{\prime }=\frac{C_{i,s}/C_{Ti,s}}{C_{i,r}/C_{Ti,r}}-1$$

In the formula, i′ represents the migration coefficient of element i in soil; C*_i,s_* and C*_i,r_* are the concentrations of element i in soil and rock, respectively; C*_Ti,s_* and C*_Ti,r_* are the contents of Ti in soil and rock, respectively. When i′ = 0, it indicates that the migration of element *i* is insignificant; when i′ > 0, it indicates enrichment of element *i*; when i′ < 0, it indicates depletion of element *i*.

#### 3.2.2. Bioavailable Fraction Ratio (F1 + F2)

The proportion of the sum of ion-exchangeable and carbonate-bound states is divided into five grades: <1% is regarded as Extremely low activity state, 1–10% is Low activity state, 10–30% is Moderate activity state, 30–50% is High activity state, and >50% is extremely high activity state.

#### 3.2.3. Secondary Phase to Primary Phase Ratio Method (RSP)

The formula is as follows:

(2) $$RSP=\frac{M_{sec}}{M_{prim}}$$

In the formula, RSP is the ratio of secondary phase to primary phase, where M_sec_ represents the heavy metal content in the secondary phase (F1 + F2 + F3 + F4), and M_prim_ represents the heavy metal content in the primary phase (F5). RSP ≤ 1 is considered primary phase dominance, 1 < RSP ≤ 2 indicates low secondary phase differentiation intensity, 2 < RSP ≤ 3 indicates moderate secondary phase differentiation intensity, and RSP > 3 is classified as intense secondary phase differentiation.

#### 3.2.4. Correlation Analysis

Normality tests indicated that some datasets deviated from normal distribution, thus necessitating the application of Spearman’s correlation analysis to explore the influencing factors of heavy metal migration and transformation. As a non-parametric testing approach, Spearman’s correlation is tailored for analyzing datasets that do not conform to normal distribution. It quantifies correlations by evaluating the rank-order relationships between variables, effectively mitigating the impact of data distribution patterns on the analysis outcomes.

## 4. Results and Discussion

### 4.1. Heavy Metal Distribution and Enrichment Patterns in Weathering Crust Profile Horizons

The mean heavy metal concentrations across different sampling horizons in the weathering crust are presented in Table 4. Significant variations are observed in the contents of eight heavy metals within the weathering crust profile of the study area. Specifically, the concentration ranges for Cr, Ni, Cu, Zn, Cd, Pb, Hg, and As are 7.96 mg/kg to 2394.95 mg/kg, 1.16 mg/kg to 696.98 mg/kg, 3.6 mg/kg to 97.54 mg/kg, 35 mg/kg to 149.95 mg/kg, 0.04 mg/kg to 0.69 mg/kg, 3.16 mg/kg to 35.86 mg/kg, 0.003 mg/kg to 0.026 mg/kg, and 0.27 mg/kg to 13.5 mg/kg, respectively. Notably, the concentrations of Cr and Ni exhibit relatively higher values, which can be primarily attributed to their association with the ultramafic rock matrix in this high-geochemical-background region. Comparative analysis with typical ultramafic rocks reveals that the ultrabasic rock weathering crust in the study area exhibits relatively higher values for Cr and Ni, while the occurrence amounts of other heavy metal elements show a relatively lower trend. This observation suggests potential hydrothermal alteration of serpentinite origin affecting the weathering crust to varying degrees. As hydrothermal fluids migrate through rock pores and fractures, they undergo geochemical interaction with host-rock minerals, leading to selective enrichment/depletion of trace elements. In the presence of hydrothermal activity, certain minerals within serpentinite and pyroxenite undergo alteration, releasing Cr and Ni from their crystal lattices into the hydrothermal fluids. Studies conducted in the gneissic terrains of western Norway have shown that this type of hydrothermal activity can promote a two-to-three order-of-magnitude increase in Cr content within the weathering crust of serpentinite compared to that in typical basic rocks. The differential mobility of metal ions during weathering is fundamentally governed by their original binding states within mineral structures [34]. As a result, in the study area, the Cr and Ni contents exhibit notably higher values compared with those in other basic rock regions. Spatial distribution patterns indicate that heavy metal contents in the weathering crust are predominantly governed by pedogenic parent materials. Meanwhile, mining activities exhibit a significant statistical association with the elevation of metal occurrence levels, suggesting a dominant role of anthropogenic factors in soil heavy metal migration and enrichment process [35].

**Figure 3:**
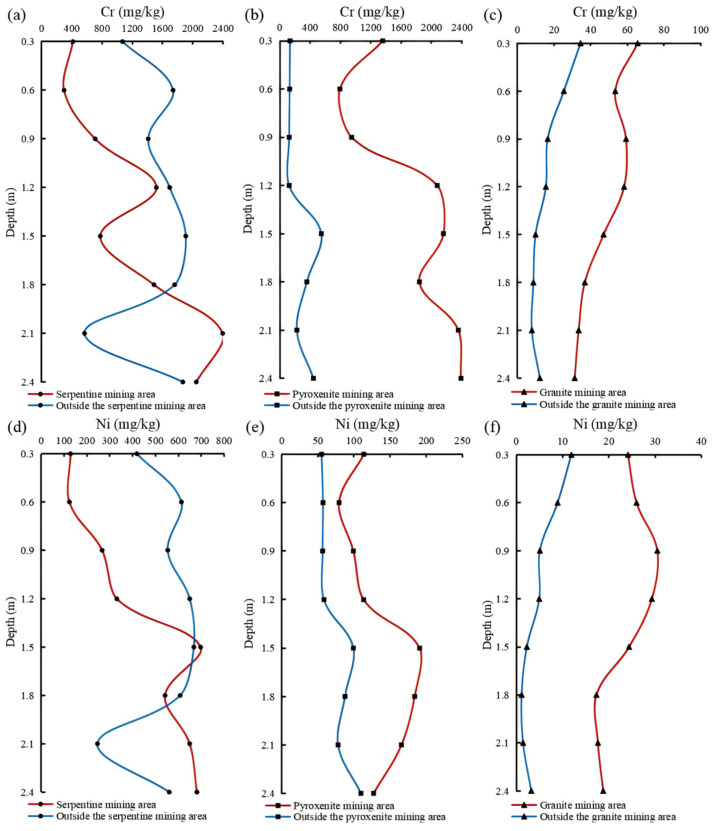
Vertical distribution of Cr and Ni concentrations in weathering crust profiles (Cr is at the top—profiles (**a**–**c**); Ni is at the bottom—profiles (**d**–**f**)).

As shown in the vertical distribution of heavy metals in the weathering crust profile (Figure 3), substantial variations in heavy metal concentrations and spatial patterns are evident across sampling sites and profile horizons. Specifically: ① Cr and Ni concentrations in serpentine and pyroxenite profiles are significantly higher than those in granite profiles, with serpentine exhibiting higher concentrations than pyroxenite. ② Vertically, Cr and Ni exhibit preferential enrichment in the saprolite zone, with a general decreasing gradient from bedrock to topsoil. Horizontally, serpentine profiles inside the mining area show lower concentrations than those outside, while pyroxenite profiles display the reverse trend. This phenomenon is attributed to hydrothermal activities during ore formation, where regions distal to ore bodies but with intense alteration exhibit elevated Cr and Ni levels. Within ore bodies, contact metasomatism between serpentine and pyroxenite drives Cr/Ni migration from serpentine to pyroxenite, resulting in inverted high-background element concentrations in the weathering crust profiles of these two mafic rocks. ③ The remaining six heavy metals show a distinct trend: concentrations decrease progressively with increasing sampling depth, accompanied by low variability. ④ Comparative results of heavy metal contents between in-mining and out-of-mining areas in granite profiles show that mining activities have contributed to the elevation of heavy metal occurrence levels, with the exception of Zn and Hg.

### 4.2. Heavy Metal Migration Characteristics in Weathering Crust Profile Horizons

**Figure 4:**
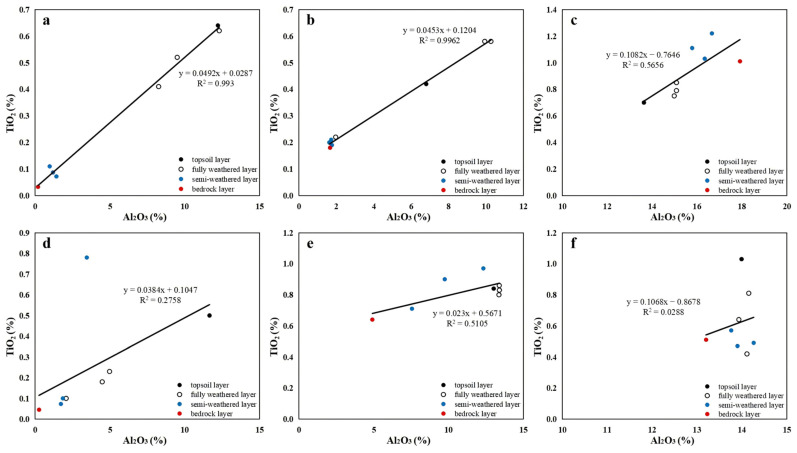
Scatter plots of TiO_2_ vs. Al_2_O_3_ in weathering crust profiles ((**a**–**c**): serpentinite, pyroxenite, and granite profiles within the mining area; (**d**–**f**): serpentinite, pyroxenite, and granite profiles outside the mining area).

As shown in Figure 4, Al_2_O_3_ and TiO_2_ exhibit strong linear correlations in weathering crust profiles within the mining area, with R^2^ values of 0.993 (serpentine), 0.9962 (pyroxenite), and 0.5656 (granite). Outside the mining area, these correlations are significantly weaker (R^2^ = 0.2758, R^2^ = 0.5105, and R^2^ = 0.0288 for serpentine, pyroxenite, and granite, respectively), indicating much better rock–soil homology within the mining area. Integrating prior analyses, hydrothermal metasomatism in the mining area restricts high-background element migration to bedrock, with no involvement in pedogenesis. Within the mining area, activities are predominantly open-pit direct mining with minimal anthropogenic interference. In contrast, indirect mining activities—such as transportation roads, tailings storage facilities, and ore dressing plants—are prevalent outside the mining area. The contrast in bedrock–soil homology between the mining area and its exterior indicates that direct mining has introduced negligible extraneous materials, whereas indirect mining activities have caused a significant influx of foreign substances. Studies demonstrate that mining-induced indirect processes, such as acid mine drainage (AMD) triggered by mining activities, can promote the migration of material components, including heavy metal elements, to surrounding water and soil media, thus inducing the phenomenon of material input [36,37,38]. Studies in the serpentine zones of Tuscany, Italy, reveal that the linear correlation between Al_2_O_3_ and TiO_2_ in soils adjacent to extra-mining transport roads is disrupted, indicating that exogenous dust has modified the material composition of the primary weathering crust [39].

**Figure 5:**
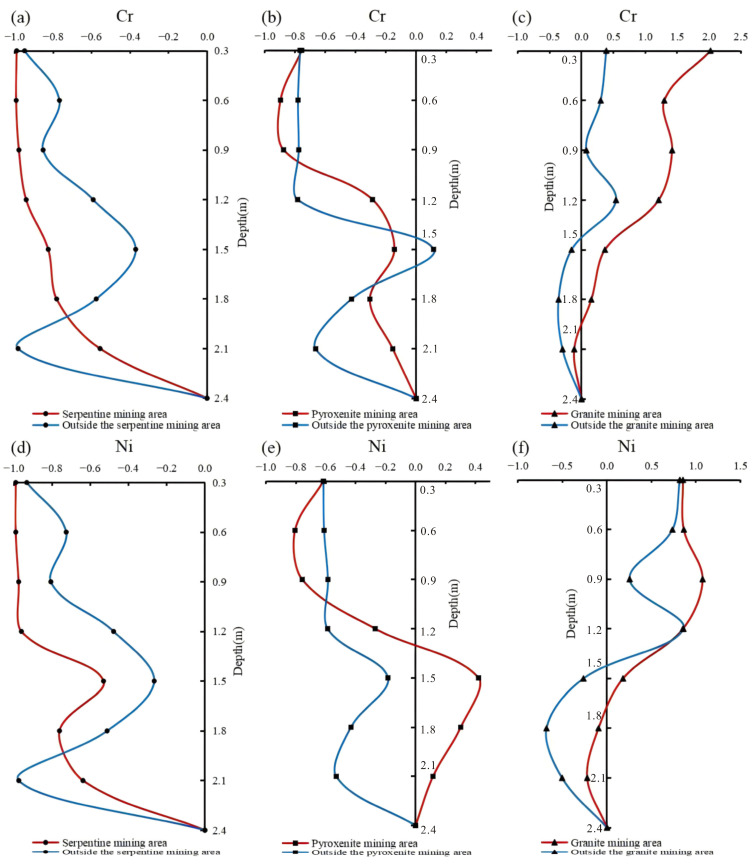
Vertical distribution of migration coefficients of Cr and Ni in weathering crust profiles (Cr is at the top—profiles (**a**–**c**); Ni is at the bottom—profiles (**d**–**f**)).

As depicted in Figure 5, Cr demonstrates overall depletion in serpentine and pyroxenite profiles. Depletion is more pronounced in serpentine profiles within the mining area compared to external areas, whereas pyroxenite profiles show the reverse trend. Notably, Cr enrichment occurs in granite profiles. Migration coefficients are overall higher inside the mining area compared to external areas, with substantial variability observed in vertical migration coefficients across the six weathering crust profiles, as detailed below: ① Within mining-area serpentine profiles, the topsoil, full-weathering, and saprolite layers exhibit profound depletion, with depletion intensity gradually diminishing from surface to subsurface. In pyroxenite profiles, the topsoil and full-weathering layers show drastic depletion, whereas depletion in saprolite layers declines abruptly. ② In extra-mining serpentine and pyroxenite profiles, comparable trends emerge: depletion diminishes gradually in horizons above the saprolite layer while increasing abruptly in deeper strata. ③ Granite profiles show consistent curve distribution patterns: migration coefficients decline progressively from surface to subsurface, with the saprolite layer exhibiting dynamic equilibrium between enrichment and depletion. Overall, Cr demonstrates limited migration and enrichment capacity during pedogenesis in mining-area serpentine and pyroxenite, whereas these processes are more pronounced outside the mining area. Indirect mining activities drive Cr enrichment in the transition zone between fully weathered and saprolite layers. Notably, higher migration coefficients in wall rocks surrounding ore-forming bodies warrant attention to the Cr excessive activity of ecological elements in this region.

Ni migration coefficient curves mirror those of Cr, with key distinctions: ① Within mining-area pyroxenite profiles, the saprolite layer exhibits enrichment, with a gradual decline in enrichment intensity. ② Granite saprolite layers uniformly show depletion. ③ Ni depletion in serpentine and pyroxenite profiles is less pronounced than that of Cr, while granite enrichment is less intense. Consequently, excessive activity of ecological elements of Ni in saprolite layers of ore-forming bedrock profiles requires prioritized attention. Parent materials exert a substantial influence on soil concentrations of Cr, Cu, Ni, and Zn [40].

### 4.3. Morphological Distribution of High-Background Elements Across Horizons in Weathering Crust Profiles

The first four fractions of the Tessier procedure are characterized by high mobility, release under mild acidic conditions, reducibility under specific redox states, and transformation to bioactive forms under specific conditions, respectively. Elevated concentrations of these fractions increase risks of secondary pollution and bioavailability, whereas the residual fraction originates from primary minerals, exhibiting chemical stability and negligible bioavailability.

**Figure 6:**
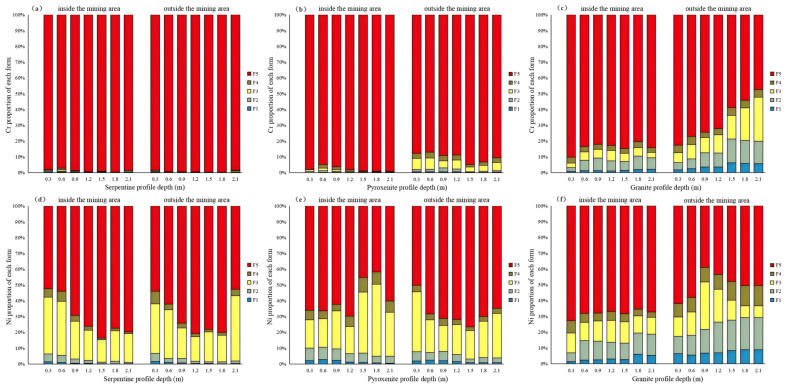
Vertical distribution of Cr and Ni speciation in weathering crust profiles. F1—exchangeable; F2—carbonate-bound; F3—Fe/Mn oxide-bound; F4—organic-bound; and F5—residual ((**a**–**c**): proportions of various speciations of Cr in serpentinite, pyroxenite, and granite profiles; (**d**–**f**): proportions of various speciations of Ni in serpentinite, pyroxenite, and granite profiles).

As illustrated in Figure 6a–c, Cr speciation reveals: ① Serpentine profiles are dominated by the residual fraction (F5), comprising 98.76% and 99.30% of total Cr within and outside the mining area, respectively. ② In pyroxenite profiles, F5 dominates within the mining area, with higher proportions in saprolite (99.11%) than in full-weathering layers (96.46%). Outside the mining area, the combined F1–F4 fraction increases to 9.76%, predominantly as F3 (5.15%), indicating Cr adsorption by Fe/Mn oxides facilitated by exogenous inputs. This results in higher F1–F4 proportions in topsoil and full-weathering layers compared to saprolite. ③ In granite profiles, F2 proportions increase notably, reaching 6.35% (mining area) and 10.37% (external area). Within the mining area, assimilation-contamination between ore bodies and wall rocks lowers F5 proportions relative to pure ore profiles. Notably, direct mining activities caused minimal exogenous input, resulting in stable speciation distributions across all horizons.

As depicted in Figure 6d–f, Ni speciation exhibits marked differences from Cr, with distinct patterns: ① Serpentine profiles in the mining area show elevated F3 (Fe/Mn oxide-bound) fractions, decreasing systematically from topsoil (5.12%) to full-weathering (2.61%) and saprolite layers (1.25%). ② Pyroxenite profiles mirror serpentine speciation trends, though with lower F5 (residual) proportions and a notable F2 (carbonate-bound) increase to 5.15%. ③ Granite profiles show F3 depletion and F2 enrichment, with higher F2 values consistently observed outside versus inside the mining area.

Collectively, these results demonstrate a significantly higher ecological element activity for Ni compared to Cr. Prior research confirms that Cr in serpentine profiles is overwhelmingly residual (>98%), posing low activity of ecological elements, whereas Ni’s elevated Fe/Mn oxide-bound fractions (2–5%) increase susceptibility to mild enrichment [41,42]. This is consistent with the present study. The excessive activity of ecological elements in weathering crust profiles decreases sequentially as granite > pyroxenite > serpentine. This is mainly because the granite is a Late Triassic intrusion into the serpentinites and pyroxenites, with higher element activity consistently observed outside versus inside mining areas.

### 4.4. Ecological Element Activity Assessment of High-Background Elements in Weathering Crust Profiles

**Figure 7:**
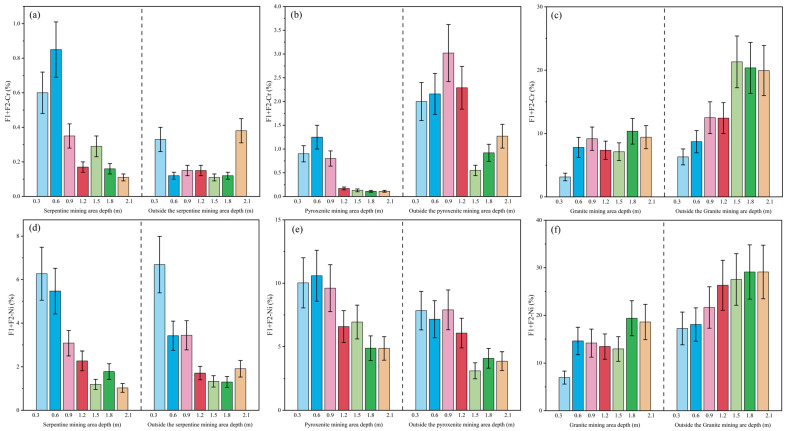
Bioavailable fraction ratio Assessment Plots for Cr and Ni in Weathering Crust Profiles (Cr is at the top—profiles (**a**–**c**); Ni is at the bottom—profiles (**d**–**f**)).

As illustrated in Figure 7, Cr and Ni show both convergent and divergent patterns in their bioavailable fraction ratio results. Convergently: ① In serpentine and pyroxenite profiles, bioavailable fraction ratio values for both elements decrease systematically with depth. ② In weathering crust profiles, Cr and Ni bioavailable fraction ratio values decrease in the order: granite > pyroxenite > serpentine. Divergences include: ① In serpentine profiles, Cr exhibits negligible element activity (mean F1 + F2: 0.28%), whereas Ni poses low element activity (mean F1 + F2: 2.92%). ② In pyroxenite and granite profiles, Cr exhibits higher bioavailable fraction ratio values outside versus inside mining areas, whereas Ni shows the reverse pattern (7.64% inside vs. 5.70% outside). For granite specifically, external bioavailable fraction ratio values (21.14%) significantly exceed internal values (14.30%).

**Figure 8:**
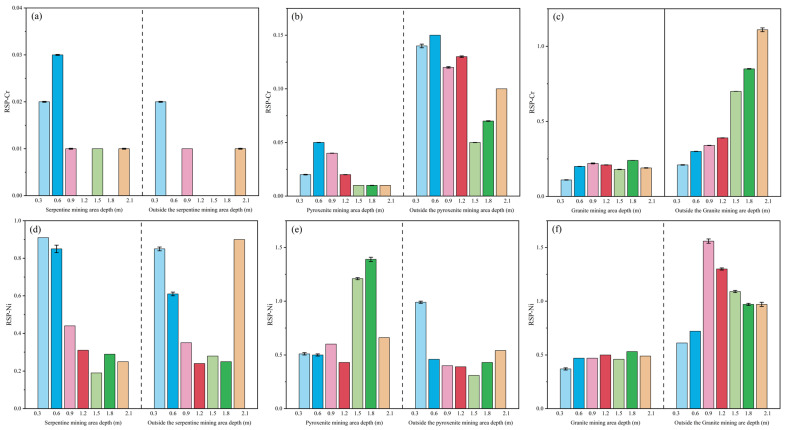
RSP Assessment Plots for Cr and Ni in Weathering Crust Profiles (Cr is at the top—profiles (**a**–**c**); Ni is at the bottom—profiles (**d**–**f**)).

Results from the RSP assessment (Figure 8) indicate that Cr and Ni are generally primary phase dominant. For Cr, low secondary phase differentiation intensity is confined to the basal saprolite of external granite profiles. Ni exhibits low secondary phase differentiation intensity in the full-weathering to saprolite transition zone of external pyroxenite profiles, with RSP values decreasing systematically with depth. In mining areas, low secondary phase differentiation intensity occurs in the upper saprolite of granite profiles.

Numerous studies highlight discrepancies in results between the two assessment methods, stemming primarily from divergent focuses and criteria: bioavailable fraction ratio evaluates bioavailable fraction element activity, whereas RSP incorporates both bioavailable and potentially available fractions. It is recommended to integrate parameters like migration coefficients and speciation differentiation indices to enhance the applicability of traditional methods (e.g., F1 + F2, RSP) in high-background regions [43,44,45,46]. Results from the two speciation-based assessments indicate that Ni poses a relatively higher ecological element activity than Cr in weathering crust profiles of the study area, with Cr showing negligible element activity. Overall, ecological element activity is higher outside than inside mining areas [47,48]. Additionally, element activity from bioavailable forms of both heavy metals decreases with increasing weathering crust profile depth.

During the evaluation of bioavailable fraction ratio and RSP, the integration of multiple indicators inherently introduces random errors into the quantification process. To mitigate this, the Monte Carlo method was implemented to simulate and characterize the impact of such errors, thereby validating the accuracy of the proposed model [49,50]. Five parameters (F1–F5) were selected as random variables, with indicator values assumed to follow a Gaussian distribution—a methodological choice well-supported for unknown real-valued distributions and extensively validated in peer-reviewed literature. The standard deviation was set at 0.1, and the effect of ±10% deviation from the initial mean values was analyzed through 1000 iterations. The Monte Carlo simulation yielded the mean values and 95% confidence intervals for F1 + F2 and RSP, as visualized by the error bars in Figure 7 and Figure 8.

Uncertainty analysis via Monte Carlo simulation indicated that the calculated actual values resided within the 95% confidence interval, with simulated means demonstrating a strong concordance with the computed results. This outcome corroborates the accuracy of the findings derived from the proposed model.

### 4.5. Influencing Factors of Heavy Metal Migration and Transformation in Weathering Crust Profiles

Profiles were stratified into two units: the soil layer (comprising topsoil and fully weathered horizons) and the parent material layer (including saprolite and bedrock).

Spearman correlation analysis (Table 5) demonstrated significant positive associations between Cr, Ni, and six other heavy metals in both soil and parent material layers within mining areas. This pattern suggests a common lithogenic origin for soil heavy metals during natural pedogenesis, predominantly derived from weathering of the underlying parent material, with no discernible anthropogenic inputs from direct mining activities. Notably, as high-background elements, Cr and Ni exhibit reciprocal promotion, while other elements exert moderate inhibitory effects, further underscoring the distinct geochemical dynamics of lithogenically enriched metals. Outside mining areas, Cr and Ni exhibited predominantly positive correlations with six other heavy metals, contrasting with patterns inside mining areas. This divergence suggests that indirect mining activities disrupted the natural evolution of heavy metals during pedogenesis. Specifically, processes such as aeolian transport of tailings, surface runoff migration, and mine drainage have introduced external heavy metals elements into surrounding soils, affecting their original occurrence states and spatial distributions [44], By analyzing correlations between Cd/Hg and Cr/Ni/Cu/Zn across soil and parent material layers, we found that indirect mining activities have promoted the input of external heavy metals elements into the soil layer, promoting Cr/Ni enrichment and resulting in higher ecological element activity outside versus inside mining areas. This aligns with Jiansheng Wu’s research, which applied ‘source-sink’ landscape and ecological element activity assessment theories to demonstrate significantly increased comprehensive ecological element activity in expanding mining zones of the Pingshuo Coal Mine District [51].

Table 6 shows consistent correlation patterns between Cr′, Ni′, and major elements in soil layers inside and outside the mining area, as follows: ① SiO_2_, Al_2_O_3_, K_2_O, and TiO_2_ exhibit positive correlations with Cr′ and Ni′. Within the mining area, correlation coefficients range from 0.425 to 0.494. Outside the mining area, significant positive correlations (*p* < 0.05) occur with the three major elements (excluding TiO_2_, which shows no significant correlation), with coefficients higher than those inside the mining area. ② Outside mining areas, Na_2_O significantly promotes Cr′ and Ni′ with correlation coefficients of 0.600 and 0.708, respectively, whereas no significant correlation exists within mining areas. Combined with observation ①, this suggests that indirect activities enhance the mobility of Cr and Ni via major elements. ③ The significant negative correlations between Fe_2_O_3_ and Cr′/Ni′ in both mining areas are attributed to the hydrolysis of iron oxides and hydroxide-rich clay minerals. As noted by Susmita Sen Gupta, clay minerals exhibit adsorption capacity for Ni, which may explain this inverse relationship [52,53,54,55,56].

Analysis of parent material layers (Table 6) reveals consistent promotion/inhibition patterns of major elements on Cr′ and Ni′, albeit with intensity variations: ① The strength of promotion/inhibition for Cr′ outside mining areas is significantly lower than within, indicating stronger control of major elements over Cr′/Ni′ in mining areas. This further supports the lithogenic homology of Cr between the parent material and soil layers in mining areas. Notably, TiO_2_ exhibits opposing correlation directions with Cr′ inside vs. outside mining areas. ② Ni′ exhibited negligible correlations with major elements outside mining areas, suggesting that indirect mining activities introduced exogenous materials, thereby impeding major elements’ control over Ni. Collectively, indirect mining activities weakened major elements’ regulatory influence on Ni significantly more than direct mining activities, while their impact on Cr was less pronounced.

Principal component analysis (PCA) (Table 7) reveals that element distribution within the mining area is predominantly governed by primary parent materials. Rock-forming elements (e.g., Na_2_O and TiO_2_) dominate the first principal component (PC1), reflecting the lithological control of parent materials, while exogenous heavy metals (Cu, Hg, As) constitute the second principal component (PC2). Notably, Cr and Ni maintain a close association with primary minerals. Outside the mining area, under the combined influence of parent material weathering and mining-induced disturbances, the coupling between exogenous elements (Cu, Hg, As, Zn) and Cr/Ni in PC2 is significantly enhanced. Mining activities promote the migration capacity of Ni active components, leading to a higher ecological element activity value of Ni (F1 + F2 = 21.14%) in the extra-mining area compared to the intra-mining area (14.30%). These findings provide robust data support for the “hydrothermal alteration–anthropogenic disturbance” dual-drive model, further confirming the distribution patterns and ecological element activity characteristics of high-background elements.

### 4.6. Regional Comparison and Countermeasure Proposals

By comparing the study area with other regions (Table 8), in ultramafic terrains, the spatial distribution of Cr and Ni concentrations and their controlling factors exhibit marked regional disparities. In Hebei’s Niangniangmiao area, serpentinite hosts Cr (417–2047 mg/kg) and Ni (129–681 mg/kg) levels significantly higher than those in pyroxenite and granite. Notably, the semi-weathered pyroxenite layer demonstrates a 1.8-fold inverted Cr enrichment driven by hydrothermal alteration. Mining activities have elevated surface serpentinite Cr by 40% outside the mining zone, introducing exogenous Zn and Cd (Spearman ρ = 0.58–0.72 with Cr/Ni), which increases the ecological element activity of Ni (RAC = 21.14%) externally versus internally (14.30%). By comparison, Poland’s serpentinized peridotites yield Ni concentrations of 1577–3944 mg/kg due to intense serpentinization, while tropical weathering in the Czech Republic’s laterite deposits fixes Cr (4500–5700 mg/kg) and Ni (3600–7700 mg/kg) in oxide phases. New Caledonia’s serpentinites, weathered under tropical climates, accumulate Ni up to 25,500 mg/kg—collectively highlighting the primacy of parent lithology, climatic regimes, and weathering intensity.

Key drivers behind these regional disparities include geological setting, climate, and anthropogenic impacts. Niangniangmiao’s “natural-human” dual-disturbance model—integrating hydrothermal alteration and mining—contrasts with single-factor systems elsewhere, such as Poland’s serpentinization degree or the Czech Republic’s tropical weathering. Notably, Ni poses higher ecological element activity than Cr in Niangniangmiao, with granite zones requiring prioritized remediation due to elevated active fractions (F1 + F2 = 15%). This case establishes a novel “hydrothermal alteration-mining disturbance” paradigm for element activity assessment, underscoring the need for site-specific heavy metals mitigation strategies tailored to geologic contexts and anthropogenic stressors.

Based on the above results, the following recommendations are provided: (1) For the semi-weathered layer of pyroxenite: Due to hydrothermal alteration forming a 1.8-fold inverted enrichment of Cr, and the Fe-Mn oxide-bound Cr (F3) increasing to 5.15% in the outer mining area, it is recommended to use chemical reduction and fixation methods (such as adding sodium sulfide) to reduce Cr(VI) to stable Cr(III), or inhibit Cr release under oxidative conditions through biofilm coverage. (2) For the granite area: The active components of Ni (F1 + F2 = 15%) have the highest proportion, and the bioavailable fraction ratio value in the outer mining area reaches 21.14%. Priority should be given to implementing phytoremediation combined with chemical stabilization: selecting Ni hyperaccumulating plants (such as the Thlaspi genus) and adjusting the soil pH to 7.0–7.5 with calcium carbonate to reduce the proportion of exchangeable Ni (F1). (3) For the serpentinite mining area: Although Cr is mainly in the residual state (>98%), mining activities have promoted a 40% increase in the occurrence amount of Cr in surface media. Soil covering and microbial improvement can be adopted to promote the transformation of Cr into an organic-bound state (F4) by adding humus, reducing biological availability.

## 5. Conclusions

(1) In the weathering crust profile, the concentrations of eight heavy metals exhibit substantial variability. Cr and Ni show significantly elevated concentrations compared to background values, with their distributions strongly influenced by pedogenic parent materials and mining activities. Cr and Ni are preferentially enriched in the saprolite layers of serpentine and pyroxenite, demonstrating a decreasing trend from bedrock to topsoil; conversely, the concentrations of the remaining six elements decline with increasing depth. Mining activities have promoted a growth trend in the occurrence amounts of most heavy metal elements, with the notable exceptions of Zn and Hg.
(2) Lithogenic homology between rocks and soils is more pronounced within mining areas than outside, suggesting that direct mining activities have not introduced exogenous materials, while indirect mining has caused a substantial material influx. Cr exhibits depletion in serpentine and pyroxenite but enrichment in granite, whereas Ni demonstrates mobility patterns similar to yet distinct from Cr. Particular attention should be paid to the excessive activity of ecological elements of Cr around ore-forming plutons and Ni in saprolite layers of ore-hosting bedrock.
(3) In serpentine, Cr is predominantly present in residual fractions, whereas in the extra-mining pyroxenite zone, Cr shows preferential adsorption by metal (hydr)oxides. In granite, the proportion of carbonate-bound Cr increases significantly. Ni exhibits distinct speciation patterns compared to Cr and presents the phenomenon of excessive activity of ecological elements. The degree of ecological element activity is granite > pyroxenite > serpentine, with external areas demonstrating higher element activity than internal mining zones. The excessive activity of ecological elements associated with bioavailable fractions decreases systematically with increasing profile depth.
(4) Within mining areas, soil heavy metals are predominantly derived from the parent material layer, where Cr and Ni exhibit mutual promotion, while other elements exert inhibitory effects on their accumulation. In contrast, indirect mining activities outside these areas disrupt elemental evolution pathways, introducing exogenous heavy metals and enhancing Cr/Ni enrichment. Specifically, indirect mining weakens the regulatory influence of major elements on Cr and diminishes their control over Ni.
(5) Optimized Translation Marked disparities exist in the impacts of anthropogenic and geological factors on metal behavior: Geologically, hydrothermal alteration drives Cr migration, climatic conditions govern Cr speciation, and weathering intensity dictates Ni release. Anthropogenically, mining introduces exogenous Zn/Cd, elevating active Ni fractions in the outer mining area. Additionally, mining-induced degradation of parent rock structures increases the migration coefficients of Cr in serpentinite and Ni in granite, thereby exacerbating ecological element activity.

## Figures and Tables

**Table 1:** List of rock unit classification and geological characteristics in the study area.

Lithostratigraphic Unit			Geological Features		
Geological time	Code	Lithology	Macrogeological characteristics	Contact relationship with the surrounding rocks	Isotopic age (Ma)
Late Triassic	ξγT_3_	Granite	The weathering is intense, with some mineral grains protruding on the weathered surface, presenting a sugary texture.	Hypabyssal intrusion νδT_3_, ψ_ω_Pt_1_, ψ_ι_Pt_1_	175 ± 10
	ξοT_3_	Syenite	Flesh-red in color, with pits on the weathered surface and scarce quartz.		235 ± 10
	ηοT_3_	Monzonite	Dark gray in color, rich in dark minerals, with scarce and unevenly distributed quartz.		
Late Triassic–Middle Triassic	νδT_3_	Diorite	Gray in color, with well-developed vein rocks.	It is hypabysally intruded by νδT_3_, ψωPt_1,_ and ψιPt_1_.	200.2 ± 2.4
Paleoproterozoic Era	ψ_ω_Pt_1_	Serpentinite	Purplish-black in color, with iron ores embedded in it.	It is hypabysally intruded by νδT_3_.	1796–1837
	ψ_ι_Pt_1_	Pyroxenite	Dark green		

**Table 2:** Analytical methods, detection limits, and quantitation ranges for parameters in weathering crust profile samples.

Indicator	Analytical Methods	Detection Limits	Indicator	Analytical Methods	Quantitation Ranges
As	Atomic Fluorescence Spectrometry	0.2 mg/kg	CaO	Total Chemical Analysis Method	0.1% to 15%
Cd	Inductively Coupled Plasma Optical Emission Spectrometry	0.021 mg/kg	MgO	Total Chemical Analysis Method	0.01% to 10%
Cr	Inductively Coupled Plasma Optical Emission Spectrometry	0.61 mg/kg	K_2_O	X-ray Fluorescence Spectrometry	0.05% to 8%
Ni	Inductively Coupled Plasma Optical Emission Spectrometry	0.6 mg/kg	Na_2_O	X-ray Fluorescence Spectrometry	0.05% to 8%
Cu	Inductively Coupled Plasma Optical Emission Spectrometry	0.6 mg/kg	Fe_2_O_3_	Total Chemical Analysis Method	≥0.05%
Pb	Inductively Coupled Plasma Optical Emission Spectrometry	0.5 mg/kg	Al_2_O_3_	X-ray Fluorescence Spectrometry	≥0.05%
Hg	Atomic Fluorescence Spectrometry	0.005 mg/kg	SiO_2_	Total Chemical Analysis Method	≥5%
Zn	Inductively Coupled Plasma Optical Emission Spectrometry	1.1 mg/kg	TiO_2_	Total Chemical Analysis Method	0.2% to 10%

**Table 3:** Procedural steps for chemical speciation analysis of heavy metals.

Analysis Steps	Speciation Analysis	Extractant	Operation Steps
F1	exchangeable	16 mL of 1 mol/L MgCl_2_ solution	Adjust the pH to 7.0, then shake continuously at 25 °C for 1 h. Centrifuge for 20 min, aspirate the supernatant, and dilute the solution to 25 mL in a volumetric flask. Wash the residue with deionized water. Following centrifugation, filter the entire supernatant and then measure the heavy metal concentration.
F2	carbonate-bound	16 mL of 1 mol/L NaAc solution	Adjust the pH to 5.0, then shake continuously at 25 °C for 8 h. Centrifuge for 20 min. Aspirate the upper supernatant and dilute to a 25 mL volumetric flask. Wash the residue with deionized water. Following centrifugation, filter the entire supernatant and then measure the heavy metal concentration.
F3	Fe/Mn oxide-bound	16 mL of 0.04 mol/L NH_2_OH·HCl and 25% (*v*/*v*) HAc mixed solution	Incubate at (96 ± 3) °C with intermittent shaking for 4 h, then centrifuge for 20 min. Aspirate the upper supernatant and dilute to a 25 mL volumetric flask. Wash the residue with deionized water. Following centrifugation, filter the entire supernatant and then measure the heavy metal concentration.
F4	organic-bound	3 mL of 0.01 mol/L HNO_3_, 5 mL of 30% (*v*/*v*) H_2_O_2_, and 5 mL of 3.2 mol/L CH_3_COONH_4_	Adjust the pH to 2 with HNO_3_, heat in a water bath to (85 ± 2) °C, and shake intermittently for 2 h. Then add 5 mL of H_2_O_2_, adjust the pH to 2 again, heat at (85 ± 2) °C for another 2 h with intermittent shaking. Cool to (25 ± 1) °C, add 5 mL of 3.2 mol/L NH_4_Ac in 20% HNO_3_ solution, dilute to 20 mL, shake continuously for 30 min, and centrifuge for 20 min. Aspirate the upper supernatant and dilute to a 25 mL volumetric flask. Wash the residue with deionized water. Following centrifugation, filter the entire supernatant and then measure the heavy metal concentration.
F5	residual	HCl + HNO_3_ + HF + HClO_4_	After digestion, transfer the solution to a 50 mL volumetric flask and dilute to the mark. Use this solution as a sample for heavy metal concentration determination.

**Table 4:** Heavy metal distribution across weathering crust profiles (data in parentheses: outside the mining area; remaining data: mining area).

Weathered Crust Type	Sampling Horizon	Data Evaluation Metrics	Cr	Ni	Cu	Zn	Cd	Pb	Hg	As
Serpentine profile	topsoil layer	Measured value (mg/kg)	417 (1072)	129 (419)	22.1 (36.8)	75.6 (129)	0.13 (0.25)	19.9 (35.9)	0.020 (0.026)	10.1 (13.5)
	fully weathered layer	Mean value (mg/kg)	842 (1615)	240 (605)	23.6 (19.2)	80.0 (122)	0.12 (0.16)	16.6 (16.0)	0.019 (0.013)	10.2 (6.7)
		Mean Relative Error (%)	53.5 (8.43)	32.5 (5.74)	2.31 (10.6)	6.66 (6.04)	2.70 (17.1)	12.4 (13.2)	4.91 (36.8)	0.98 (24.8)
	semi-weathered layer	Mean value (mg/kg)	1553 (1412)	629 (507)	23.6 (10.8)	87.1 (116)	0.07 (0.14)	5.9 (15.5)	0.009 (0.005)	2.3 (3.6)
		Mean Relative Error (%)	36.1 (39.8)	9.30 (34.3)	47.2 (5.03)	13.2 (4.25)	12.4 (70.5)	12.4 (75.3)	15.9 (13.6)	31.9 (41.9)
	bedrock layer	Measured value (mg/kg)	2047 (1869)	681 (560)	9.6 (5.9)	86.1 (99.5)	0.04 (0.06)	3.16 (6.16)	0.005 (0.005)	0.6 (2.7)
Pyroxeniteprofile	topsoil layer	Measured value (mg/kg)	1357 (135)	114 (55)	23.5 (75.2)	68.5 (119)	0.09 (0.24)	21.5 (15.6)	0.019 (0.017)	5.6 (7.4)
	fully weathered layer	Mean value (mg/kg)	1272 (126)	97 (58)	19.8 (88.6)	59.1 (139)	0.08 (0.28)	17.1 (18.7)	0.010 (0.017)	5.7 (6.9)
		Mean Relative Error (%)	42.1 (2.14)	12.4 (1.47)	33.8 (6.72)	26.4 (5.12)	15.6 (8.08)	38.0 (3.19)	33.2 (13.1)	49.8 (4.20)
	semi-weathered layer	Mean value (mg/kg)	2117 (377)	180 (88)	17.3 (75.2)	63.0 (110)	0.12 (0.21)	13.0 (14.8)	0.006 (0.013)	1.9 (5.4)
		Mean Relative Error (%)	8.70 (30.3)	5.38 (8.11)	8.70 (16.1)	13.7 (2.07)	8.96 (6.26)	17.7 (4.14)	18.8 (11.5)	13.7 (8.89)
	bedrock layer	Measured value (mg/kg)	2388 (443)	127 (110)	9.3 (25.0)	70.4 (62.7)	0.09 (0.11)	14.1 (6.92)	0.005 (0.009)	0.8 (2.9)
Granite profile	topsoil layer	Measured value (mg/kg)	66 (35)	24.1 (11.9)	21.0 (13.0)	69.0 (81.1)	0.16 (0.12)	29.8 (18.4)	0.016 (0.019)	7.7 (3.5)
	fully weathered layer	Mean value (mg/kg)	57 (19)	28.6 (6.3)	22.5 (8.9)	79.7 (90.0)	0.15 (0.10)	28.0 (17.8)	0.019 (0.013)	9.3 (1.8)
		Mean Relative Error (%)	21.4 (4.12)	27.4 (6.04)	17.8 (5.96)	1.74 (2.72)	6.00 (18.0)	2.06 (3.77)	10.3 (8.25)	33.5 (5.70)
	semi-weathered layer	Mean value (mg/kg)	39 (9)	23.7 (1.6)	18.7 (4.1)	92.3 (83.0)	0.14 (0.07)	24.8 (18.1)	0.014 (0.009)	3.8 (0.3)
		Mean Relative Error (%)	8.22 (13.4)	26.0 (18.4)	8.21 (14.9)	4.42 (9.26)	5.24 (17.6)	8.29 (5.46)	3.70 (22.6)	21.1 (33.2)
	bedrock layer	Measured value (mg/kg)	31 (12)	18.8 (3.24)	7.8 (5.1)	150 (75.7)	0.69 (0.14)	25.4 (15.2)	0.004 (0.010)	1.8 (0.4)

Note: For the topsoil layer and bedrock layer, only one sample was taken for each profile, and the numbers in the table represent the measured values of the samples; for the fully weathered layer and semi-weathered layer, three samples were taken respectively, which are presented by the mean value and mean relative error.

**Table 5:** Spearman correlation coefficients of heavy metal concentrations in weathering crust profiles (gray shading: outside the mining area).

Layer		Cr	Ni	Cu	Zn	Cd	Pb	Hg	As
soil layer	Cr	1	0.939 **	0.448	0.501	0.404	−0.269	0.136	0.739 **
	Ni	0.622 *	1	0.49	0.538	0.435	−0.189	0.05	0.622 *
	Cu	0.105	0.35	1	0.825 **	0.961 **	0.196	0.457	0.713 **
	Zn	−0.322	0.168	0.406	1	0.835 **	0.263	0.243	0.720 **
	Cd	−0.719 **	−0.26	−0.133	0.607 *	1	0.414	0.489	0.751 **
	Pb	−0.734 **	−0.853 **	−0.049	0.119	0.526	1	0.19	0.193
	Hg	−0.571	−0.305	0.014	0.406	0.587 *	0.504	1	0.529
	As	−0.3	0.462	0.24	0.656 *	0.471	−0.212	0.018	1
parent material layer	Cr	1	0.979 **	0.462	0.552	−0.245	−0.671 *	−0.278	0.531
	Ni	0.538	1	0.455	0.531	−0.259	−0.689 *	−0.216	0.538
	Cu	−0.147	−0.147	1	0.462	0.517	−0.334	0.49	0.902 **
	Zn	−0.51	−0.252	−0.364	1	0.196	0.021	0.015	0.629 *
	Cd	−0.552	−0.811 **	0.196	0.231	1	0.545	0.45	0.259
	Pb	−0.559	−0.881 **	0.308	0.217	0.902 **	1	−0.026	−0.432
	Hg	−0.385	−0.084	0.783 **	0.049	−0.035	0.14	1	0.516
	As	−0.476	−0.287	0.769 **	0.21	0.42	0.517	0.762 **	1

* *p* < 0.05; ** *p* < 0.01.

**Table 6:** Spearman correlation coefficients between heavy metal migration coefficients (Ti) and major elements in soil layers across weathering crust profiles.

Placement	Migration Factor	Layer	SiO_2_	CaO	MgO	Al_2_O_3_	Fe_2_O_3_	K_2_O	Na_2_O	TiO_2_
Inside the mining area	Cr′	soil layer	0.494	−0.304	−0.508	0.425	−0.683 *	0.449	−0.06	0.435
	Cr′	pedogenic soil matrix	0.967 **	0.05	−0.912 **	0.883 **	−0.633	0.619	0.883 **	0.883 **
	Ni′	soil layer	0.463	−0.34	−0.474	0.459	−0.642 *	0.418	−0.102	0.467
	Ni′	pedogenic soil matrix	0.483	0.517	−0.477	0.5	−0.633	−0.008	0.517	0.4
Outside the mining area	Cr′	soil layer	0.593 *	−0.24	−0.582 *	0.589 *	−0.594 *	0.583 *	0.600 *	0.085
	Cr′	pedogenic soil matrix	0.433	0	−0.433	0.45	−0.65	0.567	0.467	−0.333
	Ni′	soil layer	0.781 **	−0.088	−0.746 **	0.743 **	−0.777 **	0.742 **	0.708 *	0.217
	Ni′	pedogenic soil matrix	−0.05	0.033	0.05	0.05	−0.233	0.2	0.05	−0.283

* *p* < 0.05; ** *p* < 0.01.

**Table 7:** Loadings of heavy metals and soil property factors in weathering crusts inside and outside mining areas.

Indicator	Inside the Mining Area		Outside the Mining Area	
	PC1	PC2	PC1	PC2
SiO_2_	0.084	0.077	0.127	0.020
CaO	0.097	−0.138	−0.057	−0.168
MgO	−0.163	0.028	−0.124	−0.041
Al_2_O_3_	0.124	−0.022	0.134	0.101
Fe_2_O_3_	−0.109	−0.022	−0.118	−0.049
K_2_O	0.145	−0.071	0.124	−0.049
Na_2_O	0.257	−0.154	0.116	−0.041
TiO_2_	0.226	−0.166	0.053	0.041
Cr	−0.038	−0.049	−0.101	0.039
Ni	−0.208	0.096	−0.092	0.032
Cu	−0.093	0.345	0.001	0.261
Zn	0.044	−0.127	−0.034	0.208
Cd	0.052	−0.062	0.008	0.103
Pb	0.046	0.051	0.084	−0.150
Hg	−0.153	0.352	0.089	0.324
As	−0.177	0.423	0.003	0.313
Eigenvalue	10.808	2.953	8.827	4.727
Variance/%	60.045	15.404	49.038	26.263

Note: The rotation method used was Kaiser Normalized Varimax; the rotation converged after three iterations.

**Table 8:** Comparison of Cr and Ni concentrations in ultramafic rocks from different regions and key influencing factors.

Region	Parent Material Type	Cr Concentration (mg/kg)	Ni Concentration (mg/kg)	Key Influencing Factors	Ref.
Polymetallic Mining Areas in Southern China	Serpentinite, Peridotite	42.3–72.4	25.9–42.6	Mining activities combined with weathering of ultramafic rocks have led to significant Ni and Cr pollution in downstream farmland.	[20,44]
Hualien, Taiwan, China	Serpentinite	540	2440	Serpentinite directly weathers into high-Ni soil, and the paddy soil environment enhances Ni activity.	[44]
Eastern Mining Areas of China	Ultramafic rock contact zone	61.0–72.4	26.9–42.6	Mining disturbances accelerate the alteration of ultramafic rocks, leading to the diffusion of Ni and Cr to the surrounding areas.	[20]
Humid Areas in Asia (Japan, Taiwan of China, the Philippines, Vietnam)	Serpentinite	1000–3000	1000–2000	The higher the degree of serpentinization, the more Mg is lost, the Ca/Mg ratio increases, and the concentrations of mobile forms (PMFs) of Cr and Ni increase.	[9,34]
Poland	Serpentinized peridotite	2196–2915	1577–3944	The degree of serpentinization determines the amount of Ni released, and the surface soil is enriched in Ni due to weathering.	[18]
Red Clay Mining Area in Southern Czechia	Weathered laterite of ultramafic rocks	4500–5700	3600–7700	Tropical weathering forms laterite, and Ni and Cr are fixed by oxides.	[18]
Tuscany, Italy	Serpentinite	3502	2342	Weathering is weak in temperate climates, and Cr and Ni exist in residual forms.	[39]
The Italian Alps region	Serpentinite	1649–3428	750–2370	The concentrations of Cr and Ni are influenced by parent rock types, soil pH, organic carbon, and mineral composition.	[15]
New Caledonia	Serpentinite	>1000	10,000–25,500	In tropical climates, ultramafic rocks undergo intense weathering, and Ni and Cr are enriched through the pedogenesis process.	[13]
The Klamath Mountains, California, USA	Serpentinite	1000–4000	100–1000	In soils formed from ultramafic rocks (including peridotite and serpentinite), the distribution of Cr and Ni is controlled by topography and the degree of weathering, with mobility increasing under acidic conditions.	[13]
Galicia, Spain	Serpentinite	1499–4309	76–373	In soils formed by weathering of ultramafic rocks (serpentinite), Cr and Ni are mainly hosted in magnesium silicates and iron oxides.	[33]

## Data Availability

The data presented in this study are available on request from the corresponding authors.
